# Recent Advances in Sodium Magnetic Resonance Imaging and Its Future Role in Kidney Disease

**DOI:** 10.3390/jcm12134381

**Published:** 2023-06-29

**Authors:** Alireza Akbari, Christopher W. McIntyre

**Affiliations:** 1 Robarts Research Institute, Western University, London, ON N6A 3K7, Canada; aakbari@uwo.ca; 2 Lilibeth Caberto Kidney Clinic Research Unit, London Health Sciences Centre, London, ON N6A 5W9, Canada; 3 Departments of Medicine, Pediatrics and Medical Biophysics, Western University, London, ON N6A 3K7, Canada

**Keywords:** chronic kidney disease, hypertension, inflammation, sodium MRI, skin, heart, kidney, corticomedullary gradient, myocardium, functional sodium MRI

## Abstract

Sodium imbalance is a hallmark of chronic kidney disease (CKD). Excess tissue sodium in CKD is associated with hypertension, inflammation, and cardiorenal disease. Sodium magnetic resonance imaging (^23^Na MRI) has been increasingly utilized in CKD clinical trials especially in the past few years. These studies have demonstrated the association of excess sodium tissue accumulation with declining renal function across whole CKD spectrum (early- to end-stage), biomarkers of systemic inflammation, and cardiovascular dysfunction. In this article, we review recent advances of ^23^Na MRI in CKD and discuss its future role with a focus on the skin, the heart, and the kidney itself.

## 1. Introduction

Declining kidney function is a characteristic feature of chronic kidney disease (CKD) that leads to fluid and electrolyte imbalance, particularly sodium, in the body. High dietary salt is implicated in various diseases including hypertension [1] and cardiovascular disease [2,3]. Hypertensive individuals are at a 75% higher risk of developing CKD compared to those with normal blood pressure [4]. Furthermore, individuals with CKD face a heightened risk of cardiovascular disease and mortality [5,6]. Given the susceptibility of CKD patients to sodium-related physiological changes, understanding how sodium is managed in CKD can offer insights into the mechanisms of the disease and aid in optimizing and personalizing therapeutic interventions.

**Figure 1:**
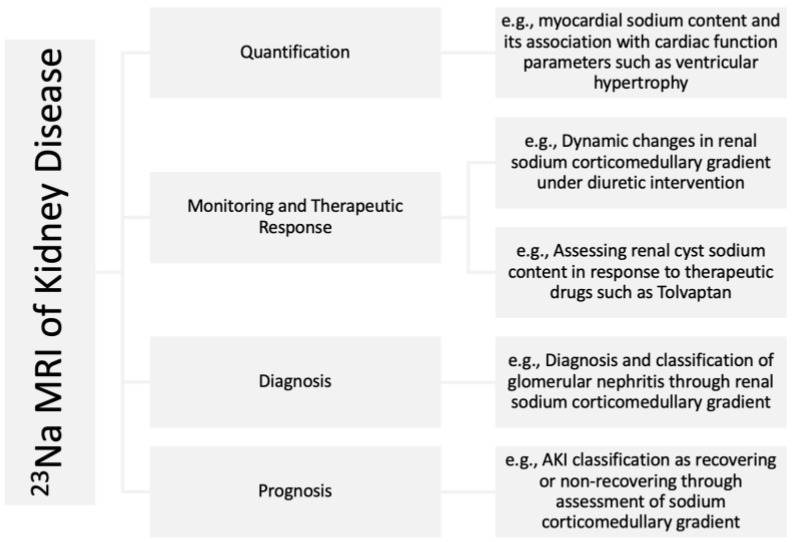
Examples of potential applications of ^23^Na MRI in kidney disease.

Techniques such as radioactive ^22^NaCl [7], ashing [8,9], and inductively coupled plasma-optical emission spectrometry [10] have been previously reported for measuring tissue sodium content. However, these techniques are not suitable for the clinical setting due to their destructive nature. Sodium magnetic resonance imaging (^23^Na MRI) allows repeated noninvasive in vivo quantification of soft tissue total sodium content (TSC) [11]. ^23^Na MRI is an increasingly utilized imaging technology that can provide a direct biomarker to assess emerging sodium removal therapeutics in CKD. Despite its first human use almost four decades ago [12], ^23^Na MRI had not been widely used in clinical research up until the past couple of decades. This has been enabled by advances in sodium imaging technology [13,14,15] that have made it possible to produce quality sodium images within acceptable signal collection times. ^23^Na MRI validity in determining TSC was demonstrated by comparing ^23^Na MRI signal to the directly measured sodium content in the muscle and skin of animals and humans using gold standard chemical analysis [16]. The first use of ^23^Na MRI in CKD research was reported around a decade ago [17]. Subsequently, its utility in CKD research has been increasingly appreciated in recent years. A comprehensive review of ^23^Na MRI in CKD has previously been provided by Martin et al. [18], augmented since by additional studies [19,20,21,22,23,24,25]. A list of studies involved in the use of ^23^Na MRI in kidney disease is summarized in Table 1. In this article, we review the past and the most recent potential clinical applications of ^23^Na MRI in CKD and discuss its future potentials (Figure 1) in managing CKD with a focus on skin, the heart, and the kidney itself.

## 2. ^23^Na MRI Evaluation of the Skin Sodium Content

Our understanding of sodium storage in the body has evolved over the past 20 years. In classical physiological teaching, ingested sodium when combined with inadequate excretion leads to expansion of the extracellular water compartment, congestion, and increasing blood pressure [35]. This model is now being augmented with additional appreciation of retained sodium being stored without associated water, within skin, muscle, and bone [16,17,36].

Skin is the largest organ in the body that provides a major extracellular reservoir for sodium storage, by providing proteoglycan chains available for sodium to bind without water [37,38]. It has previously been demonstrated that elevated sodium concentration in the skin microenvironment provides a barrier to infection [39]. A high skin sodium concentration also helps to modulate immune cell function [40,41] through enhancing proinflammatory and antimicrobial macrophage function and T-cell activation [39,42,43,44,45]. Animal studies show that interstitial sodium clearance in the skin is handled by mononuclear phagocyte system-derived vascular endothelial growth factor C-(VEGF-C-) mediated lymphangiogenesis [46,47], reducing VEGF-C bioavailability results in sodium accumulation in the skin and salt-sensitive hypertension [46,47,48].

Skin sodium content naturally increases with age. This was demonstrated by Kopp et al. [16] in healthy adults (men and women) for the first time using ^23^Na MRI, and these observations were further strengthened by subsequent studies [17,32,33]. More recently, Salerno et al. [25] have extended the range of individuals studied to include children and adolescents (healthy and with CKD). The authors reported significantly lower skin sodium content in healthy children and adolescents (age ≤ 17) compared with healthy adults. Aging is described as a pro-inflammatory process [49] that may be associated with tissue sodium accumulation due to reduced clearance of sodium by VEGF-C-mediated lymphangiogenesis. The skin sodium accumulation is aggravated in the CKD population, independent of age, worsening as kidney function declines. The first ^23^Na MRI study of the lower leg in hemodialysis (HD) showed older HD patients (≥60 years old) have significantly higher skin sodium content and lower serum VEGF-C compared with age-matched healthy controls [17]. Furthermore, recent ^23^Na MRI investigations of the lower leg in CKD (stages 3–5) [32], HD, and peritoneal dialysis (PD) patients [32,33] have revealed skin sodium concentration increases progressively across the whole CKD spectrum, with HD and PD having the highest associated sodium concentration compared to healthy controls. Moreover, Qirjazi et al. [32] reported a negative correlation between skin sodium concentration and estimated glomerular filtration rate (eGFR) in a merged control-CKD group, linking the excess skin sodium accumulation to worsening renal function. The excess skin sodium storage is also shown to be modifiable and associated with VEFG-C level. Quantitative ^23^Na MRI of the lower leg conducted by Dahlmann et al. [17] demonstrated that dialysis reduces skin sodium in HD patients. They also reported that HD patients with low VEFG-C have significantly higher skin sodium content compared with HD patients with high VEFG-C post HD. Recently, Lemoine et al. [34] demonstrated that the removal of stored sodium in the skin could further be controlled through modulating the dialysate sodium concentration. It is interesting to note that Dahlmann et al. [20] demonstrated that renal transplantation led to lower skin and muscle sodium accumulation in 31 stage-5 CKD patients receiving living-doner kidneys. They also reported improved renal function, normalization of blood pressure, and an increase in VEGF-C concentration post-transplantation.

Low-grade chronic systemic inflammation is a hallmark of CKD [50]. Markers of systemic inflammation such as interleukin 6 (IL-6), high-sensitivity C-reactive protein (hsCRP), and serum albumin have been associated with skin sodium content using ^23^Na MRI. Mitsides et al. [31] demonstrated elevated skin sodium, IL-6, and VEGF-C in stage 5 CKD patients. Similarly, Sahinoz et al. [33] reported higher IL-6 and hsCRP levels correlated with increased skin sodium content in dialysis patients. Furthermore, a negative correlation was found between serum albumin and skin sodium content in HD patients by Salerno et al. [22]. Sodium-glucose cotransporter 2 (SGLT2) inhibitors increase urinary sodium excretion by preventing sodium reabsorption in the renal proximal tubule [51]. A recent review of clinical trials has reported that SGLT2 inhibitors lower the risk of kidney disease progression and hospitalization for heart failure both in CKD and heart failure patients [52]. These results highlight the importance of the link between excess skin sodium accumulation and chronic systemic inflammation and the significance of sodium removal on improving clinical outcomes in CKD patients. Other ^23^Na MRI studies have demonstrated the efficacy of sodium removal therapeutic interventions. This was first reported over a single HD session, using ^23^Na MRI of the HD patients’ lower leg [17]. Recently, Lemoine et al. [34] investigated the effects of modulating dialysate sodium concentration on skin sodium clearance using ^23^Na MRI in the longer term (pre-dialysis state). The authors demonstrated that dialysate with a sodium concentration of 137 mmol/L (over a 12-month period) was associated with a lower amount of skin sodium compared to patients randomized to receive dialysis against a sodium concentration of 140 mmol/L. Additionally, in a case report of an 80-year-old male, Penny et al. [19] demonstrated a reduction in skin sodium content after transitioning from high-flux HD to expanded HD using a medium cut-off dialysis membrane (potentially with greater permissiveness to sodium removal compared to conventional dialysis membrane characteristics). They also reported improved depression, anxiety, and uremic pruritus scores following the switch to the medium cut-off dialysis membrane. The use of ^23^Na MRI in assessing sodium removal therapeutic interventions is increasing. Novel therapeutic approaches to sodium removal are emerging that are also amenable to the assessment of tissue sodium content using ^23^Na MRI. For example, a current clinical trial (ClinicalTrials.gov registered NCT04603014, accessed on 16 June 2023) is underway that utilizes ^23^Na MRI to assess interdialytic zero sodium containing PD fluid to augment sodium removal and ameliorate congestion in HD patients struggling with volume removal reliant on HD alone.

CKD is an umbrella term comprising a large group of heterogeneous pathophysiological conditions that affect salt balance in the body differently. As such, tubular disorders such as Bartter’s, Gitelman’s, and Fanconi syndromes are associated with kidney salt wasting. This is in contrast to proteinuric glomerular diseases, such as minimal-change disease and focal segmental glomerulosclerosis, which are associated with sodium retention [53,54]. The association between tissue sodium accumulation and CKD etiology is not very well explored. Recently, Salerno et al. [25] conducted a first-time ^23^Na MRI of the lower leg of pediatric CKD (age ≤ 17). Once the group was stratified based on etiology, the authors found high whole-leg sodium Z-scores in four patients with glomerular disease and one kidney transplant recipient due to atypical hemolytic-uremic syndrome and low whole-leg sodium Z-scores in two patients with tubular disorders.

Insulin resistance (IR) is highly prevalent in CKD and becomes almost universal towards end-stage renal failure [55,56]. IR in CKD has implications for increased cardiovascular risk factors including oxidative stress [57], chronic inflammation [57], and endothelial dysfunction [58]. The muscle is considered the primary site of IR [55]. The severity of IR has been associated with enhanced muscle catabolism in maintenance hemodialysis (MHD) patients [59,60]. Elevated tissue sodium is reported in MHD patients [17] and is shown to be even higher in HD patients with type 2 diabetes [30]. Deger et al. [29] investigated the association of lower leg tissue sodium accumulation measured by ^23^Na MRI with IR in eleven MHD patients. They reported an inverse relationship between muscle sodium content and glucose and leucine disposal rates suggesting muscle sodium content might be a determinant of IR in MHD patients. This finding highlights the fact that standard HD prescription does not successfully address the normalization of tissue sodium. Further therapeutic intervention studies are needed to investigate the effect of tissue sodium normalization on IR in CKD and particularly MHD patients.

Dietary salt intake studies reveal the contribution of high salt consumption to hypertension [1] and the increased risk of cardiovascular disease [2,3]. Sodium toxicity in CKD has been known about since 1949, when restricted daily dietary sodium intake (150 mg/day) was used to manage hypertension through the ‘Rice Diet’ experiment [61]. At the same time, hypertension, by itself, could cause CKD [4]. As shown by a meta-analysis of pooled data, the risk of developing CKD (GFR < 60 mL/min/1.73 m^2^) in hypertensive patients is 75% higher than in normotensive individuals [4]. Furthermore, excess dietary salt intake is shown to be associated with higher mortality rates in HD patients [62]. These findings are in parallel with ^23^Na MRI studies. Schneider et al. [27] reported a strong correlation between skin sodium content and left ventricular mass in CKD patients (a well-recognized risk factor for mortality). A recent study by Lemoine et al. [21] revealed that HD patients accumulate a similar amount of sodium in their skin as compared with heart failure patients, highlighting that excess sodium in HD may exacerbate cardiovascular risks in heart failure patients. Moreover, Salerno et al. [22] demonstrated for the first time the association between excess skin sodium content in dialysis patients and observed mortality.

## 3. Emerging ^23^Na MRI and CKD-Related Cardiomyopathy

The pathophysiology of hemodialysis-associated cardiomyopathy (HD-CMP) is complex. Abnormal cardiac and vascular structure along with function play a key part in excessive cardiovascular events and premature mortality experienced by patients with end-stage renal disease (ESRD) requiring HD [63,64]. Drug and device-based therapies that reduce cardiovascular morbidity in the general population have been ineffective in those receiving HD [65,66,67,68,69]. The pattern of heart disease associated with kidney failure is characterized by cardiac hypertrophy and fibrosis [70]. These two mechanisms are closely linked, as cardiac hypertrophy is associated with interstitial matrix expansion and increased expression of proteoglycans such as syndecan-4 and small leucine-rich proteoglycans [71,72] (capable of binding sequestered Na^+^, analogous to interstitial dermal storage). Sodium then potentially exacerbates this process by recruiting and activating fibroblasts yet further via tonicity-responsive enhanced binder protein activation [73,74]. Proton MRI detects signs of diffuse myocardial fibrosis that are present even from the early stages of chronic kidney disease [75]. This cardiac pathology becomes even more evident in patients receiving HD, where fibrotic features (imaged with cardiac MRI) are associated with increased left ventricular mass and serum biomarkers of cardiac fibrosis (e.g., galectin-3) [76].

Stored sodium is not biologically inert and is associated with a variety of pathophysiological consequences, including inflammation [32,33], aberrant tissue remodeling [77], hypertension [36], and fibrosis [78]. These effects are independent from the well-appreciated effects of pressure and volume overload [77,78]. Sodium MRI clinical data showing increased salt storage is observed in cardiovascular high-risk patients, e.g., CKD patients and, in particular, those requiring maintenance dialysis [17,32]. Schneider et al. reported that increased skin sodium concentration in patients with CKD is associated with left ventricular hypertrophy (LVH) independent from blood pressure and total body overhydration [27]. As suggested by Bottomley [79], increased imaged myocardial sodium signal may be the result of increased intracellular and/or extracellular sodium. Lemoine et al. [21] have recently demonstrated that heart failure exacerbates sodium storage when combined with CKD. They have shown that patients with cardiorenal syndrome have significantly higher tissue sodium concentrations than those with similar levels of renal function, but with no evidence of heart failure [21]. Parallel with these findings, a study by Friedrich et al. [23] revealed higher skin and muscle sodium content and serum IL-6 in HD patients with CVD, but with no significant difference in excess extracellular water from HD patients without CVD. Furthermore, in a more recent study, ^23^Na MRI has shown that skin tissue sodium concentration is associated with a marked increase in (predominantly cardiovascular) mortality in dialysis patients [22].

Direct evidence of toxic myocardial sodium deposition is still somewhat scant. In a small animal model looking at imaged and directly measured myocardial sodium concentration, preclinical evidence has shown that LVH and fibrosis is associated with increased intracellular myocardial sodium [80]. More recently, Christa et al. showed that increased myocardial sodium content in humans with primary hyperaldosteronism may be associated with pathways that lead to LVH [81]. The authors suggested that increased myocardial sodium signal may be the result of water-free interstitial cardiac sodium accumulation, similarly to mechanisms observed in the skin.

Direct ^23^Na MRI evaluation of myocardial sodium content could provide a previously unconsidered therapeutic target for the management of the principal cause of morbidity and mortality in patients with CKD. Understanding the role of myocardial sodium accumulation may also allow for the optimal selection of patients that might benefit from the range of emerging treatments targeting enhanced sodium removal and allow clinicians to follow the response to treatment in advance of longer term and potentially irreversible effects of tissue injury becoming apparent.

## 4. Emerging Renal Functional ^23^Na MRI

There is an urgent need to identify and develop additional techniques to diagnose kidney disease, provide patient-specific prognostic data, and follow response to treatment. There has been very little in the way of novel renal diagnostics developed within the last 20 years. Methods remain relatively crude, usually providing information averaged over the whole renal mass. Blood and urine-based methods provide limited information (especially in the non-proteinuric patient) to reliably predict outcomes on an individual basis.

Kidney function is usually assessed by the estimation or measurement of the glomerular filtration rate (GFR), focusing exclusively on the filtration process. However, kidney disease often has impacts on other components of the kidney, within the medulla, such as tubules and peritubular circulation. Many studies have shown the highly predictive value of interstitial fibrosis and tubular atrophy in CKD progression (irrespective of etiology) in both native kidney and allograft tissue-based studies [82] and the references therewithin. Corticomedullary gradient (CMG) exploration would provide a relevant assessment of tubular dysfunction independently of glomerular function and could be of significant prognostic value [83,84]. Increased medullary sodium concentration (combined with urea in the inner medulla) sets up an oncotic gradient that increases the reabsorption of water into the parenchyma, to be redistributed to the circulation [85]. Therefore, there is a physiologically significant difference in sodium concentration between the cortex and medulla. This CMG has previously been studied by invasive micro puncture experiments in rodent kidneys thus far [85,86,87]. Because of the lack of a non-invasive tool to explore this gradient in humans, all our fundamental knowledge is based on these animal studies. An imaging-based approach would provide such a non-invasive tool.

Previous animal ^23^Na MRI studies have demonstrated the feasibility to quantify sodium CMG in steady state, post renal obstruction [88], and diuretic infusion [89,90], providing tubular functional information. Dynamic measurement of sodium CMG has also been performed recently in pigs after diuretic challenge [90]. Limited studies have attempted to measure the sodium CMG in humans in steady state, post water deprivation [91], and water load [92], with variable technical success. Recently, the first application of ^23^Na MRI in CKD was reported by Akbari et al. [24]. The authors demonstrated a correlation (r^2^ = 0.22; *p* < 0.001) between sodium corticomedullary ratio and urinary osmolarity in both healthy volunteers and CKD patients with cardiorenal syndrome.

^23^Na MRI could potentially have relevant application in the following populations:

Chronic kidney disease: Recent epidemiological studies have shown significant associations with the incidence or progression of diseases including CKD and low osmolarity [83,84,93]. However, urine osmolarity is representative of overall renal mass function. The use of ^23^Na MRI could provide direct visualization and quantification of abnormalities or disruptions in sodium CMG in different regions of the kidney that may not be captured by urine osmolarity alone. Furthermore, ^23^Na MRI could be applied in assessing kidney function in ESRD where the collection of a urine sample would be challenging due to little to no remaining residual renal function (RRF). RRF is strongly associated with survival and quality of life in dialysis patients, and methods of evaluating RRF are still being refined [94].

Glomerulonephritis (GN): GN causes inflammation in the glomeruli and other parts of the kidney, and its diagnosis and classification are based on renal biopsy [95]. GN remains the second most common cause of end-stage kidney failure worldwide [95]. The renal sodium CMG may be altered since elevated tissue sodium is associated with inflammation [31,32,44]. Sodium MRI has the potential to be used as a non-invasive diagnostic and classification tool for GN, potentially rectifying the need for renal biopsy, an invasive procedure that carries risks of complications and discomfort for the patient.

Acute kidney injury (AKI): AKI leads to a sudden drop in kidney function and could be due to glomerular or tubular dysfunction [96]. Prognosis in AKI is currently difficult to predict but is very important because of the high mortality rate associated with more severe and slowly resolving AKI. In critically ill patients, the mortality was reported at 53% in the Acute Renal Failure Trial Network (ATN) study [97] and 44.7% in the Randomized Evaluation of Normal versus Augmented Level of Renal Replacement Trial (RENAL) [98]. A ^23^Na MRI of the lower leg in AKI patients revealed that skin and muscle sodium content did not change after 5–6 days of hemodialysis [28]. This may suggest that the kidney itself may be a better site for ^23^Na MRI to assess AKI in the early days of the injury. Furthermore, preclinical studies have demonstrated the utility of ^23^Na MRI in monitoring kidney recovery post AKI [99,100,101]. Maril et al. reported a 21% reduction in the sodium CMG of rats post acute tubular necrosis [99]. In a rat study, a positive correlation between total ^23^Na kidney content and fibrotic markers was reported following renal ischemia-reperfusion injury (IRI) [100]. Rasmussen et al. reported a reduction in sodium CMG in pigs post IRI [101]. It could be hypothesized that the recovery of medullary salination is a vital earlier component of renal recovery and evaluation of sodium CMG using ^23^Na MRI could classify AKI as either non-recovering or recovering.

Transplanted patients: The feasibility and usability of ^23^Na MRI of a human renal allograft was demonstrated in a single case report alone [102]. Renal transplant recipients often exhibit renal function deterioration and have a higher vasopressin concentration than healthy controls [103]. In a ^23^Na MRI study, Moon et al. reported significantly lower sodium CMG in six kidney transplant patients compared to six healthy individuals [26]. It is currently very challenging to elucidate causes of acute and chronic graft dysfunction without resorting to renal biopsy and to predict recovery in the setting of graft primary non-function. Sodium CMG measured using ^23^Na MRI may help in monitoring and predicting recovery non-invasively.

Autosomal dominant polycystic kidney disease (ADPKD): ADPKD is the most common inherited progressive renal disease caused by the mutation of two major genes, PKD1 and PKD2, and the rare genes, GANAB and DNAJB11 [104,105]. ADPKD leads to the formation and multiplication of fluid-filled renal cysts causing renal function to decline [104,105]. The management of ADPKD is limited to the treatment of symptoms and complications [106]. TEMPO and REPRISE studies have demonstrated Tolvaptan’s (TVP) ability to slow the decline in GFR and modulate changes in the total kidney volume [107,108]. A high degree of variability in TVP diuresis response has been reported in several studies [109,110,111,112]. It could be hypothesized that ^23^Na MRI might provide a therapeutic monitoring tool, through the measurement of sodium CMG, to determine the TVP dose personalized to each individual patient.

Diuretic resistance in CKD with heart failure (HF) syndrome: CKD and HF represent concurrent disease epidemics [113,114]. Both conditions have increasing incidence and prevalence in older age groups as well as persons with hypertension, diabetes mellitus, or other cardiovascular and kidney disease risk factors [115]. The presence of one condition appears to accelerate the progression of the other; having both conditions increases the risk of hospitalization, rehospitalization, the need for intensive care or kidney replacement therapy, and death [116,117,118,119,120]. The prevalence of moderate to severe kidney impairment is approximately 30–60% in all patients with HF [121]. The Acute Decompensated Heart Failure National Registry (ADHERE) database reported data on over 100,000 patients with HF requiring hospitalization, and only 9% had a normal kidney function [122]. The cornerstone of managing fluid retention in these patients is loop diuretics (used in >90% of patients) [123], with furosemide being the most common. The pathophysiology of diuretic resistance is multi-factorial and involves sympathetic nervous system activation, renin–angiotensin–aldosterone system (RAAS) activation, nephron remodeling, pre-existing renal function alterations, disrupted pharmacokinetics/pharmacodynamics of diuretics, and intravascular fluid depletion [124]. Diuretics rely on tubular delivery to have their effect, and therefore, pre-existing or worsening renal dysfunction further limits their effectiveness. A threshold effect for sodium excretion exists, requiring a minimum tubular drug concentration before effect [125]. This makes it impossible to extrapolate the response to a previous lower dose to predict the effects of a larger dose. Doses sufficient to control fluid retention and symptoms of congestion may be as low as 20 mg of furosemide, or as high as 1000+ mg per day. Dose escalation is often too slow or incomplete (resulting in catastrophic fluid retention and transfer from ambulatory care to hospital) or precipitous (resulting in excessive fluid removal and acute kidney injury), and clinical practice currently relies on ‘trial and error’. Approaches based upon monitoring urinary sodium and volume have been attempted [126,127,128]. Although several studies have illustrated the prognostic value of urinary sodium following a first administration of a loop diuretic, prognostic value during consecutive days is unproven [129] and the efficacy of subsequent doses is falling off due to the braking phenomenon and tubular remodeling [130]. The response of CMG measured by ^23^Na MRI to fluid restriction and diuretic challenge might help to guide diuretic dose prescription and subsequently improve efficacy and patient safety for the management of patients with HF and renal dysfunction. This contention is currently under clinical study (ClinicalTrials.gov registered NCT04170855, accessed on 16 June 2023).

## 5. Conclusions

In this article, we have reviewed ^23^Na MRI studies in CKD that demonstrate the association of sodium with hypertension, systemic inflammation, and cardiovascular disease. This association may point to sodium as a new therapeutic target. Fortunately, new therapies targeting sodium removal are becoming available for patients with HF and CKD, alone or in combination. In patients with at least some degree of RRF, pharmacological therapies such as SGLT2 inhibitors promote natriuresis (and deplete tissue-bound sodium pools) [131]. Sodium intake can be reduced via dietary restriction; however, this is often difficult to achieve in patients who are already severely restricted and struggling to maintain adequate nutrition [132]. Oral binders/inhibitors of absorption are under development to limit systemic exposure to ingested sodium [133]. Targeting sodium directly, rather than sodium and water together, is now becoming a therapeutic reality. Direct sodium removal (DSR) is a therapeutic application of a novel implanted instillation and drainage system, combined with 0% sodium containing PD fluid. The potential impact has already been demonstrated in large animal models and an initial study in humans with severe heart failure [134].

^23^Na MRI has been increasingly utilized in CKD clinical research especially in the past few years. Its ability to repeatedly and non-invasively quantify soft tissue TSC in the skin has been very well demonstrated, and its use in assessing myocardial and renal sodium content in the setting of CKD is emerging. The ability of ^23^Na MRI to provide a direct assessment of soft tissue TSC makes it a valuable research and clinical tool in diagnostic, prognostic, and monitoring therapeutics in kidney diseases. However, it is important to note that ^23^Na MRI is still a relatively new technique in the clinical setting. The findings discussed in this review are mostly conducted in small populations. Further large-scale clinical trials are required to validate these findings.

## Figures and Tables

**Table 1:** ^23^Na MRI studies in CKD, HD, PD, AKI, and kidney transplant.

Author	Year, Country	Study Type	Sample Size	MRI Measurement	Other Measurements
Moon C et al. [26]	2014, USA	Prospective	HC = 6; renal transplant = 6	^23^Na and ^1^H MRI of the kidney	N/A
	**Findings:** Significantly lower sodium concentration and CMG in the transplanted kidneys compared to the native kidneys				
Dahlmann A et al. [17]	2015, Germany	Cross-sectional	HC = 27; HD = 24	^23^Na and ^1^H MRI of lower leg skin and muscle pre and post dialysis	Dialysate and ultrafiltrate sodium content; VEGF-C level
	**Findings:** Increase in tissue sodium content and degrease in VEGF-C correlated to age; Higher sodium and water content in HD > 60 year and lower VEGF-C relative to HC; Post-HD, significantly higher skin sodium content found in patients with lower VEGF-C				
Schneider MP et al. [27]	2017, Germany	Cross-sectional	Mild to moderate CKD = 99	^23^Na MRI of the lower leg and ^1^H MRI of the heart	BIS; 24 h BP
	**Findings:** Skin sodium content correlated with systolic BP and left ventricular mass; Skin sodium content is a strong explanatory variable for left ventricular mass, unaffected by BP and total body overhydration				
Hammon M et al. [28]	2017, Germany	Observational	HC = 14; AKI = 7	^23^Na/^1^H-MRI of lower leg	N/A
	**Findings:** No change in muscle and skin sodium and water content following hemodialysis treatment; Muscle and skin sodium content in AKI patients significantly higher compared to HC				
Deger S et al. [29]	2017, USA	Cross-sectional	HC = 8; MHD = 11	^23^Na MRI of the lower leg	Body composition; Blood sample
	**Findings:** Lower glucose, leucine disposal rates, and higher muscle sodium content found in MHD patients; Glucose and leucine disposal rates inversely correlated with muscle sodium content in MHD patients				
Kopp C et al. [30]	2018, Germany	Prospective	HD with T2DM = 10; HD without T2DM = 30	^23^Na/^1^H-MRI of lower leg	BIS; BP; Blood sample
	**Findings:** Higher skin and muscle sodium content in HD with T2DM as compared to control HD; Excess extracellular water correlated with HbA1c; Muscle sodium content was lowered in a greater degree in HD with T2DM post-dialysis				
Mitsides N et al. [31]	2019, UK	Cross-sectional	HC = 11; CKD (stage 5) = 23	^23^Na MRI of the lower leg	BIS; 24 h BP *; Blood sample
	**Findings:** CKD patients had FO compared to HC; Higher skin but not muscle sodium content in CKD as compared to HC; Muscle and skin sodium content correlated with FO; CKD patients had elevated levels of vascular cell adhesion molecule, tumor necrosis factor-alpha, and IL-6 and lower levels of VEGF-C; FO in CKD linked to higher IL-8 and inversely associated with E-selectin; Higher skin sodium linked to higher ICAM				
Qirjazi E et al. [32]	2020, Canada	Cross-sectional	HC = 10; CKD = 12; HD = 13, PD = 10	^23^Na MRI of lower leg	Blood sample
	**Findings:** Elevated skin, soleus, and tibia sodium content in HD and PD patients; Negative correlation between serum albumin and soleus muscle sodium in HD; Negative correlation between eGFR and sodium content in combined HC-CKD; Negative correlation between hemoglobin and sodium tissue concentration in CKD and HD				
Sahinoz M et al. [33]	2020, USA	Cross-sectional	HC = 119; PD = 10; HD = 33;	^23^Na MRI of lower leg	Blood sample
	**Findings:** Significantly higher skin and muscle sodium in HD and PD compared to HC; Higher muscle and skin sodium content in African American patients on dialysis compared with non-African Americans; Skin and muscle sodium content correlated with age; Higher skin sodium content in male sex; Higher ultrafiltration correlated with lower skin sodium in PD; Higher skin and muscle sodium content correlated with greater plasma IL-6 and hsCRP levels; Higher variability in tissue sodium content in repeated scans from patients with higher sodium in their baseline scan				
Penny J et al. [19]	2021, Canada	Case report	An 80-year-old male	^23^Na MRI of lower leg	Depression score; Anxiety score; Pruritus score
	**Findings:** Reduction in soft tissue sodium in the lower leg along with lower depression, anxiety, pruritus scores after therapy				
Lemoine S et al. [34]	2021, Canada	Cross-sectional	HD = 18 with [Na^+^]_D_ = 137 mmol/L; HD = 18 with [Na^+^]_D_ = 140 mmol/L	^23^Na MRI of lower leg	Blood Pressure
	**Findings:** Significantly lower skin sodium content in HD group on [Na^+^]_D_ = 137 mmol/L prescription; Skin sodium content correlated with systolic blood pressure				
Dahlmann A et al. [20]	2021, Germany	Prospective	HC = 31; CKD (stage 5) undergoing kidney transplant = 31	^23^Na MRI of lower leg	BIS; BP; Blood and urine samples
	**Findings:** Higher skin and muscle sodium content in CKD compared to HC; No difference in plasma sodium concentration between the two groups; Lower skin and muscle sodium content after kidney transplant was associated with improved renal function, normalization of blood pressure and increase in lymphatic growth-factor concentration				
Lemoine S et al. [21]	2021, Canada	Cross-sectional	HF = 18; HD = 34; CKD = 31	^23^Na MRI of lower leg	Blood sample; Urine sample; Echocardiography
	**Findings:** Indistinguishable skin sodium content between the HF and HD groups; Significantly higher skin and muscle sodium content in HD than CKD patients; Significant correlation between skin sodium and urinary sodium; Lower skin sodium content in patients who were volume depleted (sodium excretion fraction <1%) than patients with sodium excretion fraction >1%				
Salerno F et al. [22]	2022, Canada	Observational	42 HD = 42; PD = 10	^23^Na MRI of lower leg	Blood sample
	**Findings:** Skin sodium content associated with all-cause mortality and composite events, independently of age, sex, serum sodium and albumin; Dialysate sodium, serum albumin and congestive heart failure significantly associated with skin sodium content in HD patients (R^2^adj = 0.62)				
Friedrich AC et al. [23]	2022, Germany	Observational	HD with CVD = 23; HD without CVD = 29	^23^Na MRI of lower leg	BIS; BP; Blood sample
	**Findings:** Higher skin and muscle sodium content and inflammation marker interleukin-6 in HD patients with CVD; No significant difference in excess extracellular water between both groups				
Akbari A et al. [24]	2022, Canada	Exploratory prospective	HC = 10; CKD = 5	^23^Na MRI of the kidney	Urine sample
	**Findings:** Reduced kidney sodium medulla-to-cortex ratios and mean urinary osmolarity from fasting baseline to peak urine dilution after water-load in healthy volunteers; Kidney sodium medulla-to-cortex ratio and corresponding urinary osmolarity correlated in both groups (r^2^ = 0.22; *p* < 0.001)				
Salerno F et al. [25]	2023, Canada	Case-control exploratory	Pediatric HC = 17; Pediatric CKD = 19; Adult HC = 19	^23^Na MRI of lower leg	Blood sample; Urine sample
	**Findings:** Significantly higher tissue sodium content in healthy adults compared to pediatric groups; No significant differences between pediatric HC and CKD groups; High whole-leg sodium concentration Z-scores in four patients with glomerular disease and one kidney transplant recipient due to atypical hemolytic-uremic syndrome; Reduced whole-leg sodium concentration Z-scores in two patients with tubular disorders; Proteinuria and hypoalbuminemia significantly associated with tissue sodium concentration				

* Abbreviations: CKD, chronic kidney disease; HD, hemodialysis; PD, peritoneal dialysis; AKI, acute kidney injury; HC, healthy control; CMG, corticomedullary gradient; VEGF-C, vascular endothelial growth factor C; BP, blood pressure; BIS, bioimpedance spectroscopy; T2DM, type 2 diabetes; HbA1c, glycated hemoglobin; FO, fluid overload; IL, interleukin; ICAM, intracellular adhesion molecule; eGFR, estimated glomerular filtration rate; hsCRP, high-sensitivity C-reactive protein; [Na^+^]_D_, dialysate sodium concentration; CVD, cardiovascular disease.

## Data Availability

Not applicable.
